# Pt(IV) Complexes as Anticancer Drugs and Their Relationship with Oxidative Stress

**DOI:** 10.3390/biomedicines13040981

**Published:** 2025-04-17

**Authors:** Vlad Iova, Radu Ciprian Tincu, Ioana Scrobota, Mihail Silviu Tudosie

**Affiliations:** 1 Faculty of Medicine, “Carol Davila” University of Medicine and Pharmacy Bucharest, 020021 Bucharest, Romania; vlad.iova2022@stud.umfcd.ro (V.I.); radu.tincu@umfcd.ro (R.C.T.); mihail.tudosie@umfcd.ro (M.S.T.); 2 ICU II Toxicology, Clinical Emergency Hospital, 014461 Bucharest, Romania; 3 Department of Dental Medicine, Faculty of Medicine and Pharmacy, University of Oradea, 1st Decembrie Street, 410073 Oradea, Romania

**Keywords:** oxidative stress, cancer, Pt(IV), anticancer prodrugs, glutathione, ascorbic acid

## Abstract

Despite continuous research, cancer is still a leading cause of death worldwide; therefore, new methods of cancer management improvement are emerging. It is well known that in the pathophysiology of cancer, oxidative stress (OS) is a significant factor. Nevertheless, there is currently no quick or easy way to identify OS in cancer patients using blood tests. Currently, in cancer treatments, Pt(IV) complexes are preferred to Pt(II) complexes in terms of adverse effects, drug resistance, and administration methods. Intracellular reductants convert Pt(IV) complexes to their Pt(II) analogs, which are Pt compounds with anti-carcinogenic effects. Our aim was to find out if Pt(IV) complexes could be used to assess blood oxidative stress indicators and, consequently, monitor the development of cancer. In this review, we analyzed previous research using the PubMed and Google Scholar public databases to verify the potential use of Pt(IV) complexes in cancer management. We found that two main serum antioxidants, glutathione and ascorbic acid, which are easily measured using conventional methods, react favorably with Pt(IV) complexes. Our research results suggest Pt(IV) complexes as therapeutic anticancer drugs and potential diagnosis agents. However, further research must be conducted to verify this hypothesis.

## 1. Introduction

Cancer is the second leading cause of death globally after cardiovascular disease [1], according to estimates being responsible for 9.6 million deaths, or 1 in 6 deaths, in 2022 [2].

At present, cancer therapies imply different methods that, besides their advantages, often come with disadvantages like high invasiveness, postoperative morbidity and mortality, non-specificity, toxicity to the surrounding organs and tissues, drug resistance, limited data regarding the right dose, poor bioavailability, and high costs, respectively [3,4,5,6,7,8,9,10,11,12,13,14].

Although therapeutic approaches have been developed over time, cancer incidence and mortality have not decreased [15]. Therefore, the improvement of knowledge on the etiopathogenetic mechanisms of cancer, early diagnosis techniques, and screening methods could contribute to better prevention and management in already-diagnosed cases.

The development of cancer is linked to genetic dysregulation (acquired or congenital), tumor cells that bypass host immune responses, metabolic alterations and epigenetic modifications like DNA hypermethylation and hypomethylation, histone acetylation and methylation, and dysregulation of non-coding RNAs (ncRNAs) [16,17,18]. It is well studied that oxidative stress (OS), by intracellularly generating reactive oxygen species (ROS), plays a crucial part in the development and evolution of cancer by disrupting genomic stability and signaling pathways in the cellular environment [19]. In addition, correlations between genetic dysregulation and epigenetic modifications, and OS were found. Within the body, oxidative load is modulated by the equilibrium between the genes that code for prooxidant enzymes that control processes producing oxidant species, while the genes that code for antioxidant enzymes regulate reactions involved in scavenging these species. Various genetic variations can act upon both the expression and the action of the encoded proteins, and afterwards modify their modulation of the redox processes, augmenting the OS [20]. Moreover, increased ROS levels have been proven to be implicated in the modifications of DNA methylation levels [21,22]. On the other hand, DNA methylation modifications may control the expression of genes that are related to the OS [22]. The promoters of genes that directly express oxidative or antioxidant enzymes are impacted by histone methylation, which also indirectly has significant stimulatory effects on cardiovascular OS-signaling networks [23]. Moreover, it is accepted that elevated OS is involved in increased histone acetylation [24]. Previous studies showed that ROS can enhance or reduce the expression of microRNA (miRNA) and by modulating the targeted genes, influence cellular functions [25]. Growing recent data confirms miRNAs and components of redox signaling interactions [26,27], and miRNAs as being influenced by the expression of ROS regulators and redox detectors, the key components of cellular antioxidant machinery [28].

OS is implicated in all stages of carcinogenesis and free radicals (FRs) have been described as both initiating and promoting factors of the carcinogenic process. Current studies incriminate FR in the malignization of benign tumors (i.e., tumor progression), therefore causing the final stage of carcinogenesis [29]. The processes and pathways implicated in OS are intracellularly conserved. ROS can advance many steps of tumor formation and evolution starting with the proliferation of cells and continuing with the avoidance of apoptosis or anoikis, invasion of tissue and metastasis into the extracellular matrix (ECM), mesenchymal–epithelial transition factor (Met) overexpression, and Rho-Rac interplay, and angiogenesis [30]. Concerning cellular proliferation, OS influences several biochemical processes that involve proteins important in signaling [31,32,33,34].

Researchers, through the years, have been studying the mechanism of OS installation and the way OS is implicated in the initiation and progression of pathologies, emphasizing the production and effects of FR [35]. Recent studies show that the OS does not result only from ROS, which explains why certain treatments that target only FR are inefficient [36,37,38]. Thus, the imbalance between the prooxidant and antioxidant activities of the organism should be considered in terms of antioxidant level modification as well [38,39,40]. Higher ROS levels trigger OS-induced tumor cell death, promoting anti-carcinogenetic signaling, according to multiple studies, confirming the significance of antioxidants. It was found that by producing more antioxidant proteins in response to the increasing ROS levels, cancer cells developed a detoxification system that preserved protumorigenic signaling and cell death resistance [41].

The current diagnosis of many types of tumors employs imaging techniques [42,43,44,45,46,47], correlated with the pathological assessment of biopsies and other biochemical markers [48,49,50,51,52,53,54]. Since OS is implicated in the pathogenesis of cancer, OS biomarkers could facilitate diagnosis, prognosis, and treatment approaches, and be useful in supplementing the present screening of the disease progression and treatment response for cancer patients [55]. The main OS biomarkers are the products of oxidative reactions of proteins, nucleic acids, lipids, and uric acid, and, in equal importance, the levels of the antioxidant capacity of biofluids in the human body [56].

The antioxidant systems of the organism are represented by enzymes such as catalase, superoxide dismutase, and glutathione peroxidase; macromolecules such as ferritin, albumin, and ceruloplasmin; and an array of small molecules, including α-tocopherol, β-carotene, reduced glutathione (GSH), ascorbic acid (AA), methionine, uric acid, bilirubin, and ubiquinol-10 [57]. GSH, a tripeptide with the γ-glutamylcysteinylglycine sequence, is usually the dominant intracellular thiol, with a concentration of up to 8 mM [58,59]. One of the most important functions of the thiols is maintaining the intracellular redox equilibrium of oxidized thiol/hydropersulfide [58,59]. In addition, these thiol groups containing molecules are susceptible to autoxidation catalyzed by metallic ions such as Cu(II) and Fe(III) [60,61,62]. The rates of these reactions increase with the increase in the pH [63].

L-ascorbic acid’s (L-AA) redox reaction is fundamentally important in chemistry, biochemistry, pharmacology, and other medical disciplines. L-AA is employed as a reducing agent that can cede either one or two electrons in chemical and biological systems [64,65,66,67,68,69,70].

There is a high [71,72,73,74] number of studies that prove the capacity of the coordination complexes of some metals to analyze OS. A recent study by E. Kim et al. demonstrated that K2IrCl6 can be used in serum assays related to the chemical information of OS [75]. Current research emphasizes the influence of other trace elements in modulating OS, indicating their potential beneficial effect on cancer prevention and management. According to a 2024 study, prostatic cancer patients’ survival was positively impacted by increased serum levels of both Zn and Se through the regulation of antioxidant enzyme systems [76]. The authors proposed Zn and Se levels as biomarkers of prostatic cancer progression, if not directly then through impacting signaling pathways and oncogenes, and conjugating proteins, respectively [76]. In addition, other recent findings suggested that Zn and Cu play a dual role in redox signaling and oxidative stress modulation. Therefore, optimizing Zn and Cu levels was proposed as a preventive approach for people with breast cancer gene 1 mutations. Although Zn or Cu level alone did not significantly correlate with overall cancer risk, the Zn/Cu ratio was suggested as a valuable biomarker for cancer prevention in this high-risk population [77,78].

Square-planar Pt(II) compounds have gained interest because of their remarkable anticancer properties [79,80,81]. But because Pt(II) has a number of serious side effects (such as ototoxicity, nephrotoxicity, hepatotoxicity, gastrointestinal problems, hair loss, or anemia), Pt(IV) complexes have shown a lot of interest in overcoming these drawbacks [82]. Anticancer properties were proved for Pt(IV) compounds with a potential biological activity [81]. Beyond the reduced toxicity, these Pt(IV) complexes have additional benefits: their octahedral structure permits the addition of functionalized axial ligands, which improves cellular uptake, tolerance in biological media, and selectivity against tumor cells [83,84]. They are also more chemically inert to biomolecules of the human body due to their resistance to ligand substitution. Thus, in order to act as anticancer drugs, Pt(IV) compounds must be first reduced to their Pt(II) analogs [85]. The Pt(IV)-based complex’s activity against tumors is probably owed to the efficiency of their transport intracellularly, followed by their reduction to the more therapeutically efficient Pt(II) compounds [85]. In addition, these octahedral Pt(IV) compounds are chemically inert regarding substitution reactions [85]. Also, these Pt(IV) coordination complexes are reduced to their Pt(II) analogs by different cellular reductants [86].

We aimed, therefore, to review some of the most medically significant properties of Pt(IV) compounds, emphasizing their use in cancer management as potential serum OS biomarkers and anticancer treatment agents.

## 2. Methodology

In this review, we analyzed previous studies employing the PubMed database and Google Scholar database using the following combinations of keywords: (“Pt(IV)” OR “platinum(IV)” OR “redox probing”) AND (“oxidative stress” OR “antioxidants” OR “glutathione” OR “ascorbic acid” OR “anticancer” OR “enzyme-like activity”), “Pt(IV)” AND “reduction” AND “monitoring”, “Pt(IV)” AND “prodrugs” AND “anticancer agents” AND “cytotoxic compounds”, “oxidative stress” AND “peroxidation” AND “oxidative stress-mediated apoptosis” AND “cancer”, “platinum” AND “chemotherapy” AND “mitochondrial dysfunction” AND “oxidative stress”, “anticancer drugs” AND “cancer pain” AND “pathophysiological mechanisms”, (“glutathione” OR “ascorbic acid”) AND (“cancer initiation” AND “cancer progression” AND “cancer therapy”). The database was searched for papers published between 1965 and 2024.

In vivo and in vitro studies were included, as well as reviews on the use of metal complexes in assaying the antioxidant capacity of the serum, reactions between Pt(IV) compounds and the main components of the antioxidant defense system, the physical and chemical properties of the most widely used Pt(IV) complexes in medicine, the use of these compounds in the treatment of tumors, and on the influence of GSH and AA in cancer development.

## 3. Cancer and OS

**Figure 1:**
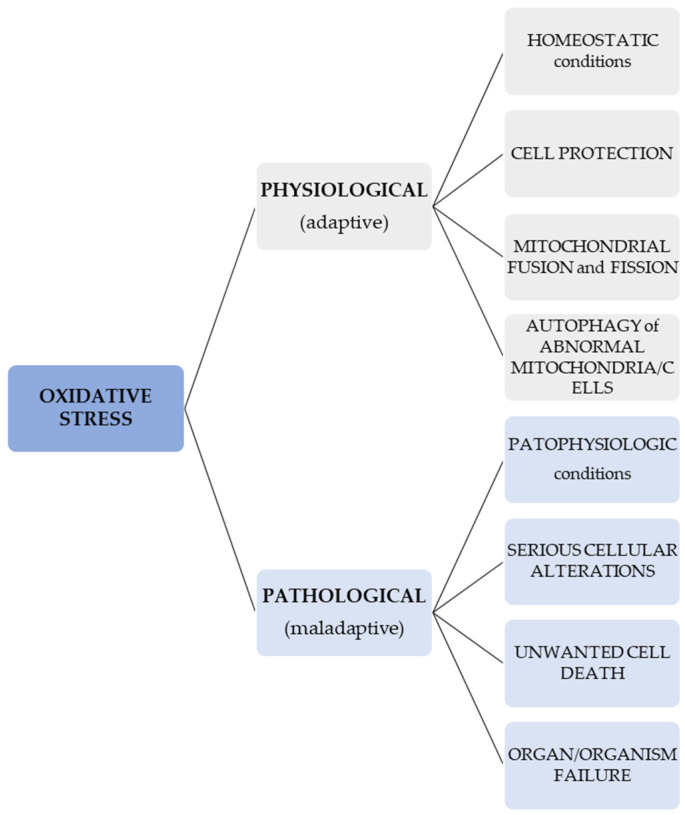
Effects of physiological and pathological OS.

In normal conditions, OS signals may lead to different cellular responses which result in the protection, fusion, and fission of mitochondria and autophagy of atypical cells or mitochondria in order to protect other cells or mitochondria [87]. On the contrary, an OS that is unregulated and pathologic may cause serious cellular alterations and unwanted cell death, and thus, can lead to organ and organism failure [88,89]. In other words, a difference ought to be considered when talking about an adaptive (physiological) redox response/stress—for instance, in the case of programmed elimination of damaged cellular systems (such as the one represented by a mitochondrion)—and a maladaptive, pathological OS [90]. This information regarding the different effects of OS is presented in Figure 1.

Physiologically, 2% of the oxygen used by mitochondria is consumed in the generation of ROS. However, if the mitochondrial antioxidant defense systems are affected, this proportion may vary from 0.25% to 11% in association with the animal species and rate of respiration [89].

Thus, ROS are implicated in maintaining normal cellular milieu but when deregulated, different diseases can occur, including cancers [41]. Increased ROS levels disrupt cellular environment equilibrium and lead to cellular function alterations, possibly contributing to the onset of various types of cancer [19].

Cancer cells typically display redox imbalance in comparison with healthy cells because of the increased metabolic rate, accumulated mitochondrial dysfunction, high cell signaling, and fast peroxisomal activities [91].

Given the latest developments, the role of ROS in cancer biology is a developing field [92]. They are thought to be typical byproducts of a variety of biological processes [92] and, when in low concentrations, were found to be implicated in angiogenesis, invasion, cell migration, and proliferation [92]. On the other hand, high concentrations of ROS can alter organelles, membranes, lipids, proteins, and nucleic acids by causing DNA mutations and pro-carcinogen generations, ultimately leading to cell death [93]. The dysregulation of Nuclear factor-kappa B (NfkB), Nuclear factor erythroid 2-related factor 2, phosphatidylinositol 3-kinase/protein kinase B/mammalian target of rapamycin, mitogen-activated protein kinase (MAPK), p53, retinoblastoma 1 gene, p21, adenomatous polyposis coli gene, tumor suppressor genes, and cell type transition signaling pathways, followed by the alteration of cyclic adenosine monophosphate, responsive element binding protein, c-Myc, c-Jun, and Fos proto-oncogene (c-fos) signaling molecules, results in improper DNA repair processes, unchecked cell proliferation, and cell death avoidance, all of them being hallmarks of cancer [19,94]. In the attempt of overcoming ROS-dependent cell death avoidance, the different mechanisms of cell death like apoptosis (i.e., cell death driven by an intracellular suicide program [95]), ferroptosis (i.e., an iron-dependent, non-apoptotic cell death caused by lipid ROS accumulation [96]), necroptosis (i.e., regulated necrosis driven by death receptors, interferons, toll-like receptors, intracellular RNA and DNA sensors, and other mediators via the proteins receptor-interacting protein kinase-3 and mixed lineage kinase domain-like protein [97]), and autophagy (i.e., an intracellular degradation through a lysosome-dependent regulated mechanism [98]) were studied [92].

Moreover, various plant-based and synthetic antioxidants to balance the carcinogenetic effects of ROS were researched. They were found to impact cancer progression, proliferation, and invasion with less adverse effects on the other cells when compared to conventional approaches [94].

## 4. Pt(IV) Compounds and Their Antitumor Activity

**Figure 2:**
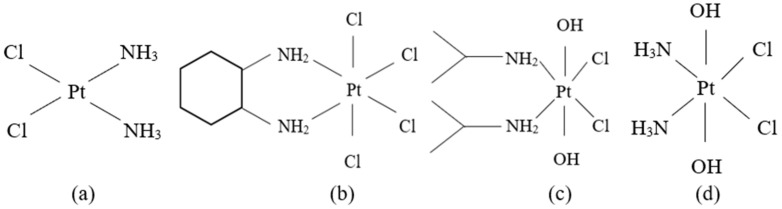
The structures of Pt anticancer drugs: (**a**) cisplatin; (**b**) tetrachloro(DL-trans-1,2-diaminocyclohexane)platinum(IV) or ormaplatin/tetraplatin; (**c**) cis,cis,trans-[Pt(*i-*PrNH_2_)_2_Cl_2_(OH)_2_] or iproplatin; (**d**) cis,cis,trans-[Pt(NH_3_)_2_Cl_2_(OH)_2_] or oxoplatin.

Pt-based anticancer drugs have been quickly clinically applied given their wide and very effective antitumor properties [99]. The identification and characterization of cisplatin, cis-[Pt(II)(NH_3_)_2_Cl_2_] ([PtCl_2_(NH_3_)_2_] or CDDP) (Figure 2a), a Pt(II) complex, was an important breakthrough that stimulated the interest in Pt(II)- and other metal-containing compounds as potential anticancer agents [100,101]. Although Pt-based anticancer drugs are the most recommended ones in several cancers, they have disadvantages like toxic adverse effects and even acquired resistance for some primary tumors [102]. As a result, intracellular reduction of Pt(IV) to Pt(II) species was seen as an encouraging approach for exerting anticancer activity and avoiding the toxic side effects of classic Pt anticancer drugs [103].

Even though the research interest moved to Pt(IV) prodrugs, most of them were only tested in vitro and only a few entered (pre)clinical trials, e.g., satraplatin. Satraplatin, one of the most promising Pt(IV) prodrugs, was found to be reduced quite rapidly in the bloodstream and was eventually discarded during the phase III clinical trial since it did not show a convincing benefit in terms of overall survival with respect to cisplatin and carboplatin [104,105]. Even though the resistance of Pt(IV) compounds to reduction is to be achieved in order to limit toxicity, these prodrugs have to be reduced at a specific moment in order to act on the cellular DNA and cause cancer cell death. Obtaining the ideal stability level, which lowers the toxicity but maintains the anticancer activity, is still challenging [106]. Further investigations in clarifying the pharmacokinetics, metabolism, and catabolism mechanism of Pt(IV) compounds, and the heterogeneity of Pt(IV) compounds are mandatory before their clinical applications as variations in chemical properties and ligand designs can affect their stability and activity [107]. Nevertheless, this field of research continues to be of great interest as these Pt(IV) compounds are regarded as an alternative to side effects, drug resistance, and the delivery way of divalent platinum drugs [107].

Exploring patient-specific variability in response to Pt(IV)-based treatments, an article from 2020 presented the potential relationship of various genetic polymorphisms in response to Pt-based chemotherapy for several types of cancer. The results revealed that through various mechanisms, genes such as Excision Repair Cross-Complementation group 1 (ERCC1), ERCC2/xeroderma pigmentosum complementation group D (XPD), xeroderma pigmentosum complementation group C (XPC), and xeroderma pigmentosum complementation group A (XPA) have been associated with patient response to Pt-based chemotherapy. According to the study’s findings, genetic polymorphism analysis is advised for the treatment of cancer to recommend treatment for each patient according to their unique genetic profile for a successful and efficient result [108].

Several Pt(IV) diamino complexes, namely tetrachloro (DL-trans-1,2-diaminocyclohexane) platinum(IV) or ormaplatin or tetraplatin (Figure 2b) [109,110,111], cis,cis,trans-[Pt(i-PrNH_2_)_2_Cl_2_(OH)_2_] or iproplatin (Figure 2c) [112,113,114,115] and cis,cis,trans-[Pt(NH_3_)_2_Cl_2_(OH)_2_] or oxoplatin (Figure 2d) [114,116,117] are the subject of current research. Nevertheless, Pt(IV) compounds can undergo ligand substitution which is much more difficult in comparison to their Pt(II) analogs, usually with an excess of either the ligand or Pt(II) being required [118]. Thus, Pt(IV) complexes are reduced to Pt(II) before the reaction with the DNA [110,112,114,119,120] and it appears that cell thiol group-containing biomolecules and AA are the most important agents of the reduction. The redox mechanism of this reduction is poorly understood, even though there are several preliminary kinetic data that have been published [41,110,112].

### 4.1. Monitoring the Reduction of Pt(IV) Complexes: Detection Principles

As high levels of ROS are associated with the initiation and progression of various types of malignancies [19], measuring the level of intracellular antioxidant agents could be a useful tool to assess cancer development. There are several tests used to determine the antioxidant activity of different samples [72]. There are methods based on the transfer of a hydrogen atom, like the Oxygen Radical Absorption Capacity (ORAC) test, the Hydroxyl Radical Antioxidant Capacity (HORAC) test, the Total Peroxyl Radical Trapping Antioxidant Parameter (TRAP) test, and the Total Oxyradical Scavenging Capacity (TOSC) test. There are also methods based on electron transfer, such as the Cupric Reducing Antioxidant Power (CUPRAC) test, the Ferric Reducing Antioxidant Power (FRAP) test, and the Folin–Ciocalteu test. Other methods are the ones that combine both hydrogen atom and electron transfer: the 2,2′-Azinobis-(3-ethylbenzothiazoline-6-sulfonic acid (ABTS) test and the [2,2-di(4-tert-octylphenyl)-1-picrylhydrazyl] (DPPH) test [72].

A review from 2024 summarized recent advances regarding the reduction profiles of Pt(IV) complexes to Pt(II) ones. They underlined that in the activation of Pt(IV) prodrugs, which are highly promising candidates for next-generation anticancer drugs [121], of great importance is the reduction chemical reaction that ultimately results in Pt exerting its anticancer effects. The strategies used to monitor this reaction are also used to predict the anticancer performance of Pt(IV) complexes since cell reductants like AA or GSH reduce Pt(IV) prodrugs intracellularly to their Pt(II) equivalents in order for them to have anticancer therapeutic valencies [121].

The reduction of Pt(IV) prodrugs to their Pt(II) analogs involves a change in the coordination geometry of the Pt center, causing a rearrangement of the electrons surrounding it that can be used to monitor this reduction process. This change in geometry occurs because the Pt(IV) complexes, presenting a low-spin configuration because of their high crystal field splitting energy and a d^2^sp^3^ hybridization, have an octahedral geometry, while the Pt(II) compounds tend to have a square-planar geometry with a low-spin configuration because the crystal field splitting energy is higher than the electron-pairing energy [121,122,123,124].

Using ultraviolet-visible spectroscopy (UV-Vis) in monitoring the reduction of Pt(IV) complexes is based on the observation that Pt(IV) complexes show a more intense and red-shifted *ligand-to-metal charge transfer* (LMCT) band than their Pt(II) counterparts [83,125]. When activated, axial ligands are lost. Therefore, the reducing intensity of the LMCT band in Pt(IV) compounds will be observed and assayed at a specific wavelength and temperature [126]. The findings of a study made by Choi et al. [127] revealed that the reduction reaction rate of the Pt(IV) compounds was influenced by two factors: the electron-withdrawing power of axial ligands and the steric hindrance of axial and equatorial ligands. Hence, the larger the complex, the shorter the time of reduction. Even though UV-Vis spectroscopy offers many advantages in observing the reduction of Pt(IV) complexes, there are also some disadvantages. The method cannot be used in living cells because of their abundance in UV-Vis absorptive proteins, nucleic acids, and pigments [121].

Another method of Pt compound detection is ^195^Pt NMR spectroscopy since the most abundant isotope of Pt is ^195^Pt (33.85%). The range of chemical shift for Pt (δ^195^Pt) is as wide as 13,000 ppm [128]. As a consequence, as the chemical shift difference between Pt(II) and Pt(IV) can be as large as thousands of ppm, this wide range allows the identification of these oxidation states (+2, +4). Furthermore, changes in the ligands attached to the Pt center can modify the value of the chemical shift by as much as 100 ppm [129]. So, ^195^Pt NMR spectroscopy is used for structural identification, comparison of oxidation states, and recognizing the coordination environment of different Pt complexes. Moreover, several databases of δ^195^Pt values can be used to monitor the reduction process from Pt(IV) prodrugs to Pt(II) drugs. ^195^Pt NMR spectroscopy can detect Pt in various chemical and coordination conditions, identify different substances containing Pt in situ, and assess chemical reactions in fluids [121]. However, one of the disadvantages of this method is that relatively large amounts of samples are required due to its low sensitivity (10 mM of sample/600 MHz NMR) [130,131]. Moreover, the Pt peaks in the spectrum may appear broadened, resulting in a more difficult analysis [128].

Due to their accessibility, the ^195^Pt NMR and UV-Vis spectroscopy methods may, in the future, be used in clinical applications not only to evaluate Pt(IV) treatment efficacy in terms of Pt(IV) reduction but also to estimate the levels of GSH and AA. Corroborating these data with other OS indicators in a larger context offered by different biochemical cancer biomarkers and clinical parameters could highlight the complexity of the carcinogenesis mechanisms and possibly contribute to clarifying some of them.

### 4.2. Pt(IV) Compounds in the Treatment of Cancer

The discovery of Pt(II)-based antitumor compound cisplatin in the 1960s represented a breakthrough in the field of oncology [132]. As of date, no other non-Pt metal-based compound has been able to successfully pass all the steps of clinical trials. The research of these complexes is of great interest, with the goal of discovering Pt compounds that have high anticancer potential, minimal undesirable side effects, potential against chemotherapy-resistant tumors, and high bioavailability [133].

In spite of their kinetic inactivity and less adverse effects, Pt(IV) complexes can undergo “in-cell” reduction, activation of the Pt center, and the generation of the therapeutic Pt(II) complexes [134,135]. The possibility to target cancerous cells could be regarded as another Pt(IV) complex advantage. The redox disequilibrium in cancer cells due to increased metabolic rate, accumulated mitochondrial dysfunction, high cell signaling, and fast peroxisomal activities may trigger these complexes to reduce them to their anticancer active counterpart [135].

Nowadays, cisplatin remains a crucial component of chemotherapy in many solid tumors such as advanced ovarian cancer, testicular cancer, bladder carcinoma, and hematologic malignancies [136,137]. Though, physicians do occasionally prescribe cisplatin for several other cancers when the benefits exceed the risks of drug side effects [138]. For instance, breast cancer, cervical and endometrial carcinoma, and gestational trophoblastic neoplasia are usually treated with cisplatin in combination with taxane derivatives, fluorouracil (5-FU), and doxorubicin [139,140,141]. Other off-label treatments include metastatic, advanced, and refractory cancers like Hodgkin lymphoma, non-Hodgkin lymphoma, penile cancer, thymoma, head and neck cancers, osteosarcoma, multiple myeloma, and mesothelioma [142,143,144].

In the attempt to avoid the adverse effects of cisplatin, such as nephrotoxicity, ototoxicity, hepatotoxicity, and gastrointestinal toxicity [100,145], another two Pt(II)-based drugs were studied and approved worldwide, oxaliplatin and carboplatin, respectively [134]. Furthermore, the researchers focused on the effects of Pt(IV)-based compounds for better specificity and fewer side effects, aspects limited in Pt(II)-based compounds treatment [146,147,148,149,150,151].

#### 4.2.1. Pt(IV) Compounds’ Mechanism of Action

Pt(IV)-based compounds do not react with biomolecules till proper conditions converge to reduce them to the corresponding Pt(II) counterpart that reacts with the DNA and restores the original reactivity. Moreover, during this reduction reaction, the axial ligands of the Pt(IV) molecule with octahedral geometry are detached and may exert additional pharmacological actions [135,146,152,153,154,155,156,157,158,159,160,161,162]. This reduction process is possible in the presence of high levels of cellular reductants (e.g., GSH) produced to overcome OS generated by the overproduction of ROS in tumor cells [163]. The two main widely accepted cellular reductants that aid the process of reduction of Pt(IV) prodrugs to Pt(II) molecules are GSH and AA [159,162,164,165,166,167].

#### 4.2.2. Structure and Ligands Depended Pt(IV) Prodrugs Properties

The octahedral Pt(IV) complex has two types of ligands: equatorial and axial ligands. The equatorial ligands are responsible for the effects of the resulting Pt(II) molecules. The axial ones, however important, are accountable for the chemical stability of the complex and for its lipophilicity and redox properties [135,168,169,170,171]. Lipophilicity ensures the entry of the molecule inside the cell through passive diffusion, determining the quantity of prodrug that enters the tumor cell, while the redox properties control the rate of reduction in the hypoxic environment of the tumor cell [170,171]. The conjugation of Pt(IV) complexes with large organic ligands leads to a relevant water solubility reduction [134]. The most studied axial ligands that influence this reduction have been chlorides, hydroxides, and carboxylates [172]. It has been reported that chloride ligands favor this reaction the most [127]. The reduction potentials are more negative when the axial ligands are represented by hydroxide and, as a result, the complexes having axial hydroxide ligands are less susceptible to reduction [172]. Regarding axial carboxylate ligands, the measured reduction potentials of their complexes are intermediate between those of hydroxide- and chloride-based compounds, and so is their reduction susceptibility [172].

**Figure 3:**
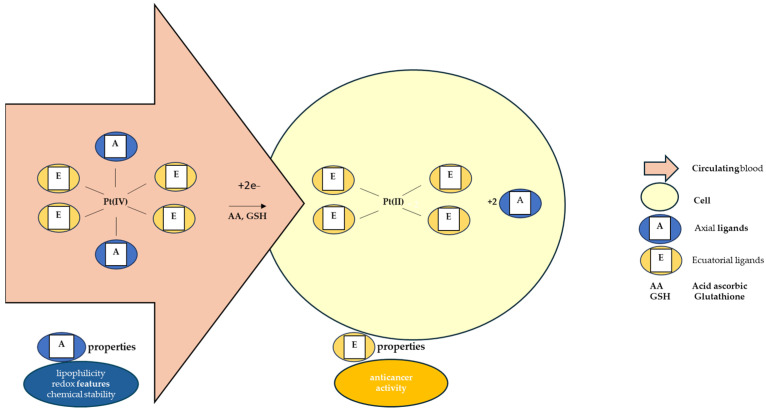
Pt(IV) intracellular reduction mechanism and properties given to the prodrugs by the type of ligands.

As Figure 3 shows, after the intracellular reduction of Pt(IV) prodrug, the free axial ligands are released in the cytoplasm, acting as possible secondary drug molecules in a synergic manner with the Pt(II) products. This principle accounts for the development of new Pt(IV)-based “combo drug” candidates [173].

As these prodrugs have six ligands around the Pt center, Pt(IV) compounds with various ligands have been proposed. In addition, different types of functionalization of the axial positions of these complexes are currently being studied, with results in reducing side effects and increasing the therapeutic efficiency of Pt chemotherapy when both cytotoxic and non-steroidal anti-inflammatory drugs are added to the Pt(IV) compounds [174]. Pt(IV) prodrugs functionalized with agents with high antitumor potential, such as tubulin polymerization inhibitors, resulted in Pt(IV) complexes that attack both DNA and tubulin, improving anticancer effects. Pt(IV) prodrugs can also be functionalized with analogs of micronutrients that are normally found in the organism like α-tocopherol succinate and α-TOS, and target both DNA and mitochondria [134].

Until now, the only Pt(IV) prodrugs introduced in clinical trials are ormaplatin (or tetraplatin), LA-12, iproplatin, and satraplatin [134].

#### 4.2.3. Reduction of Pt(IV) and Anticancer Activity

In the case of Pt(IV) prodrugs, the reduction step is of utmost importance in exerting their anticancer activity. A study analyzed the reduction reaction rates and cytotoxicity of several Pt(IV) complexes [127,130]. The rates of reduction and the reduction potentials of Pt(IV) compounds having ethylenediamine (en) carrier ligands were positively correlated with the electron-withdrawing power of the axial ligands (cis,trans,cis-[Pt(en)(OH)_2_Cl_2_] < cis,trans,cis-[Pt(en)(OCOCH_3_)_2_Cl_2_] < Pt(en)Cl_4_ < cis,trans,cis-[Pt(en)(OCOCF_3_)_2_Cl_2_]). In addition, the compounds with en carrier ligands had a lower reaction rate than their analogs with isopropylamine (ipa) and cyclohexylamine (cha) carrier ligands [127]. An interesting finding was that there was no strong correlation between reduction reaction rate and cytotoxicity towards cisplatin-sensitive cells regarding all the analyzed complexes [127]. Nevertheless, when only the compounds bearing en ligands were compared, the cytotoxicity was positively correlated with the reduction rate [127] In addition, there was also a positive correlation between cytotoxicity and reduction rate for complexes having different carrier ligands, but the same axial ligands [127].

Though, there are some cases in which the high rate of reduction does not lead to higher antitumoral activity. For example, the low reduction potential of tetraplatin causes its early reduction in blood prior to cellular uptake, lowering the drug efficiency [135,175]. On the other hand, a slow reduction step does not always lead to hindered anticancer activity. For instance, even though iproplatin is eliminated mostly unchanged through urine, the presence of traces of its Pt(II) products of metabolism shows that its reduction can occur [112,135,167].

### 4.3. Antioxidant Enzymes-like Activity of Pt

Cells are equipped with antioxidant systems to counteract OS or reduce the damage produced by ROS or their byproducts [176]. Firstly, and most effectively, the antioxidant enzymes get into action. Secondly, the exogenous intake of small molecules with antioxidant properties can step in and thirdly, oxidized products may be repaired or eliminated by antioxidant enzymes [177].

Two of the most important enzymes involved in the first-line defense mechanism are catalase (CAT) and superoxide dismutase (SOD) [177]. CAT is involved in antioxidant defense by interacting with cellular hydrogen peroxide (H_2_O_2_) to produce water and oxygen [176], while SOD is a metalloenzyme that plays a major role by catalyzing the conversion of ROS-like superoxide anion free radical (^•^O_2_^−^) H_2_O_2_ and molecular oxygen (O_2_) [178].

Carbon nanodots were functionalized with Pt nanoparticles (PtNPs) in an attempt to create a new nanozyme. The resulting Pt/carbon nanodots nanomaterial exhibited both SOD- and CAT-like specific activities of 12,605 U/mg and 3172 U/mg, respectively, with SOD activity being simulated by carbon nanodots and CAT by nanoparticles. Due to their enzyme-like activity, they were proposed as effective antioxidative stress agents [179].

Taking into consideration that the reduction reaction of Pt(IV) may be monitored by different means [121], the levels of GSH and AA, the main components of the antioxidant defense of the organism with reducing activity [57], can be evaluated using their reaction with Pt(IV) compounds.

## 5. Pt-Based Compounds, OS, and Cell Death

These results highlight the importance of understanding how trace elements could contribute, some of them both as diagnostic and therapeutic agents, to maintaining cellular homeostasis and mitigating disease progression.

Pt/MgO nanoparticles, when used in the treatment of cancer, revealed that their anticancer properties can be linked to the generation of OS and programmed cell death [180]. The production of ROS induces lipid peroxidation which can affect the phospholipids of the plasma membrane, leading to damage to the cell integrity, especially if these ROS are reactive hydroxyl radicals [181,182].

Pt-based drugs cause OS by increasing the generation of ROS [183] and by decreasing the production of GSH [184], after which apoptosis will occur through p53, survivin, Bcl-2–associated X protein (Bax)/B-cell leukemia/lymphoma 2 protein (Bcl-2), and caspase pathways [180].

Cell-death signaling takes place through two pathways: mitochondria-mediated and death-receptor-mediated, which activate caspase-9 and caspase-8 [185,186]. Caspase-3, a primary executioner caspase [187], is a downstream caspase that is involved, in both pathways, in the generation of apoptosis [180]. The above-mentioned study has shown that treatment with a precursor of GSH may undo the effects of caspase-3 which is activated by Pt/MgO nanoparticles in the tumoral cells and, thus, can inhibit apoptosis [180].

The study mentioned previously stated that treatment with Pt/MgO nanoparticles resulted in an increase in the permeability of the mitochondrial membrane and, as a result, in a decreased level of anti-apoptotic Bcl-2 and increased concentrations of pro-apoptotic Bax and p53 [180]. These modifications eventually led to the activation of apoptotic pathways [180].

Pt-based chemotherapeutic agents are used in the treatment of many types of cancer. Although otherwise efficient, they have important drawbacks related to the dose in which they can exert their anticancer potential. One of the most distressing conditions is peripheral neuropathy. It is caused by oxidative/nitrosative stress arising during chemotherapy with a 19–85% prevalence [188,189]. Altered mitochondrial function and the generation of OS were implicated as important in the occurrence of Pt-induced neuropathy [189]. These compounds enter the cells, after which they bind the mitochondrial DNA, forming adducts that cannot be repaired as there is no system to repair the DNA inside the mitochondria. These abnormal adducts affect the normal DNA replication and transcription, thus generating abnormal mitochondrial proteins, leading, consequently, to an altered respiratory chain in this organelle [90,190,191,192,193]. This affected mitochondrial function leads to a decrease in cell metabolism, greater production of ROS, and, as a result, OS [194,195,196,197]. Oxaliplatin, a Pt-based anticancer drug, was proven to remarkably increase the production of superoxide anions and to cause lipid peroxidation and the oxidation of proteins and DNA in sciatic nerves and the spinal cord [198]. The inhibition of physiological cell metabolism and increased ROS levels can cause damage to enzymes, structural proteins, and lipids, resulting in modifications in the structure of peripheral nerves [199]. ROS may induce apoptosis in nerve cells, thus leading to atrophy and loss of dorsal root ganglial cells [200,201]. Even though results from in vitro studies or research on laboratory animals presented a hypotrophy of the dorsal root ganglia together with nerve cell atrophy, magnetic resonance imaging examinations in patients treated with oxaliplatin showed hypertrophy of the dorsal root ganglia [202]. These apparently contradictory results may be due to differences in the moment of analysis and the period of time after the administration of the Pt-based oxaliplatin [189].

It was believed that the blood–brain barrier halts the entry of oxaliplatin into the brain [203]. Nevertheless, chemotherapy-induced blood–brain barrier alteration may be caused by pro-inflammatory cytokines, ROS, or neurotransmitters, which, as a result, could be involved in peripheral nervous system toxicity induced by antitumor drugs [204,205]. In a study from 2018, in vitro treatment with oxaliplatin caused important modifications in the junctional complexes and cytoskeletal system in endothelial cells associated with the blood–brain barrier, and this damage can result in greater levels of oxaliplatin in the central nervous system, and, in addition, chronification of the pain [206]. A 2016 study by Sanna et al. [207] evaluated the neuronal alterations induced by oxaliplatin administration in spinal and supraspinal levels by monitoring protein expression in the mouse cortex, thalamus, periaqueductal gray matter, and spinal cord. The authors showed that protein alterations were because of the neurotoxicity induced by oxaliplatin and reported a direct correlation between structural changes in the central nervous system and chemotherapy-induced neurotoxicity.

OS can cause neurologic damage in several diseases including diabetes, acrylamide-induced neuropathy, and Charcot–Marie–Tooth disease [208,209,210,211]. Besides the formation of Pt-DNA cross-links, it has been revealed that Pt-based drugs also produce FR, causing, as a result, oxidative damage to the DNA [212]. Several authors have reported that OS damage to nuclear DNA and mitochondrial DNA may lead to programmed cell death in nerve cells because of the Pt chemotherapy [197,213].

As evidence of the increased interest in Pt complexes and in parallel with the research on their stability, action mechanisms, and effects, the elaboration of the incorporation and delivery formulas have experienced development as well. In Pt(IV)-based therapies, nanoscale drug delivery systems have been utilized to load Pt agents, such as Pt(IV) prodrugs, to form Pt nanoparticles through physical encapsulation, chemical crosslink, or conjugation [214]. There are some nanoparticles designed to deliver Pt(IV) complexes, including carbon nanotubes, carbon nanoparticles, gold nanoparticles, quantum dots, upconversion nanoparticles, and polymeric micelles [83]. The latest developments of Pt(IV)-prodrug nanotherapeutics are micelles, nanoassemblies, polyphenol-Fe^3+^ nanoparticles, graphene oxide nanosheets, liposomes, and lipid nanoparticles [215].

## 6. The Influence of GSH in Cancer Development

GSH is the most representative intracellular antioxidant [216]. It preserves the redox equilibrium within the cells through different mechanisms such as scavenging ROS and, also participating in cell signaling through thiol exchange with S-Nitrosoglutathione and by modulating protein functions [217].

GSH displays either anti- or pro-carcinogenic effects on different types of malignancies [216]. For example, during progression, GSH was found to be essential for the elimination and detoxification of carcinogens. Changes to this pathway can significantly impact the survival of cells. But, it was reported that in bone marrow, breast, colon, throat, and lung malignancies, high GSH levels in tumor cells can shield those cells by giving them resistance to several chemotherapeutic medications [218,219].

ROS, themselves, have different effects on tumor initiation and progression when in different concentrations complicating the impact of GSH during the cancerization process. In a stressed tumor milieu, moderate ROS levels can promote survival and proliferation by triggering signaling pathways that may aid in tumor development, on the one hand. On the other hand, high levels of ROS buildup, inadequate scavenging systems, or a lack of antioxidants cause biomolecules to be severely damaged, which leads to cell death. In conclusion, there is a need for a specific, complex condition of the redox system in order for the cancer cells to live or die [216].

The effect of GSH on OS and cancer initiation and progression is also complicated because of the dual role of ROS in these processes. Moderate ROS levels can support survival and proliferation by activating signaling pathways that can contribute to tumor growth in stressful tumor microenvironments. However, excessive ROS accumulation, failure of proper scavenging mechanisms, or antioxidant scarcity results in severe damage to biomolecules, triggering cell death. Therefore, cancer cells need to maintain an intricate balance of antioxidant levels to survive. Furthermore, ROS can control the ability of tumoral cells to spread to other areas [216]. This was corroborated by data showing that both liver cancer and melanoma metastasize when GSH levels are high [220,221]. These findings highlight the complementary functions of GSH and ROS in the development and spread of cancer [222].

Besides the synthetic pathway described above, cells can convert glutathione disulfide (i.e., the oxidized form of glutathione) (GSSG) to GSH [223] activated by GSH reductase, which uses Nicotinamide adenine dinucleotide phosphate (NADPH) as a substrate, thus complicating the mechanisms by which GSH participates in carcinogenesis. Tumoral cells’ high GSH/GSSG levels could be a result of NADPH generation via the pentose phosphate pathway [224,225]. Data supporting this supposition show that clear cell renal cell carcinoma tumors have a notable build-up of increased GSH and pentose phosphate pathway-related metabolites, correlating with an enhanced pentose phosphate flow [184]. Lung cancer and melanoma were two other types of malignancies in which GSH was found to be overexpressed [224,226,227], more frequently in patients experiencing cancer recurrence after surgery [227].

Given the important role of GSH in cancer development, different therapeutic approaches to modulate its metabolism have been researched over the years. The attempts to target only GSH by reducing it or interfering with its synthesis process by modifying parameters like cystine/glutamate exchanger, glutaminase, and glutamate cysteine ligase were not successful [228,229] due to the very complex mechanisms of adaptation of the cellular antioxidant defense [229]. Nevertheless, future research to explore and exploit the need for tumors for enhanced GSH will result, eventually, in targeted cancer treatments [230].

## 7. The Influence of AA in Cancer Development

AA is one of the most important vitamins functioning as an antioxidant and anti-inflammatory agent [231]. In the body, AA is present in ascorbate form and is not able to be produced by the organism; it must be provided from exogenous sources [232].

Various mechanisms by which AA could exert its anticancer effects were considered, including depleting ROS, selectively generating ROS, stimulating their toxicity towards cancerous cells, inhibiting glucose metabolism, modulating epigenetic factors, and regulating the expression of hypoxia-inducible factor in tumor cells [233].

A critical factor of AA efficiency was proven to be an adequate interaction of the antioxidant with the cancer cells [234], therefore elevated doses were used [235]. Nevertheless, several clinical studies on cancer patients reported high AA doses, alone, or in association with other classical drugs to have reduced treatment toxic side effects and raised patients’ well-being [236,237].

As a result of previous treatment encouraging results, AA analogs have been proposed starting from substitution to the C2, C3, C5, and C6 positions of AA [238,239] and up to 2,3-O, O-dibenzyl-6-(4-decyl-1,2,3-triazol-1-yl)-6-deoxy-L-ascorbic acid [240], 6-[4-(4-bromophenyl)-1,2,3-triazol-1-yl]-6-deoxy-L-ascorbic acid [241], 2-O-α-D-Glucopyranosyl-6-O-(2-Pentylheptanoyl)-L-ascorbic acid [242], dibenzyl (S)-1-[(R)-3,4-bis(benzyloxy)-5-oxo-2,5-dihydrofuran-2-yl]-2-hydroxyethyl phosphate [243].

Both AA and its analogs are seen today as possible candidates in cancer treatment management, firstly because of their therapeutic effects and secondly, but not ultimately, because of their easy accessibility from exogenous sources, reduced side effects, and minimum costs [233].

## 8. Overview of the Reactions of Pt(IV) Compounds with GSH and AA

Rosenberg and colleagues reported in the first reports of cisplatin’s anticancer effects that some complexes containing Pt(IV) have strong antitumor activity. Since Pt(IV) complexes are slow to exchange their bound ligands, a key question regarding the mechanism of action of these compounds is how they may be activated in vivo to produce their antitumor effects. It was proposed that Pt(IV) complexes may be reduced to Pt(II) complexes in vivo, and that these complexes exert their cytotoxic effects in a manner similar to that of cisplatin and its analogs [244].

Both AA and GSH, found in blood plasma and cytosol, are small molecule-reducing agents often cited in connection with the reductive activation of Pt(IV) complexes [164]. Pt(IV):GSH stoichiometry in the reduction of trans-[PtCl_2_(CN)_4_]^2−^ by thiols was proved to be 1:2 [63]. In addition, K. Lemma et al. proved, by ^1^H-NMR spectrometry, that GSH is oxidized to GSSG in a 1:2 Pt(IV):GSH mixture, at 25 °C and pH approximately 7, thus attesting the 1:2 stoichiometry of the reduction reaction [86]. Moreover, U. S. Mehrotra et al. demonstrated that the stoichiometry of the reduction reaction of Pt(IV) by AA is 1:1 [245].

### 8.1. Reaction of Pt(IV) with GSH

A 2019 study analyzed the reaction of carboplatin Pt(IV) prodrug cis,trans-[Pt(cbdca)(NH_3_)_2_Cl_2_] (cbdca = cyclobutane-1,1-dicarboxylate) activation of the by the major small-reductive molecule reductants in blood [246].

**Figure 4:**
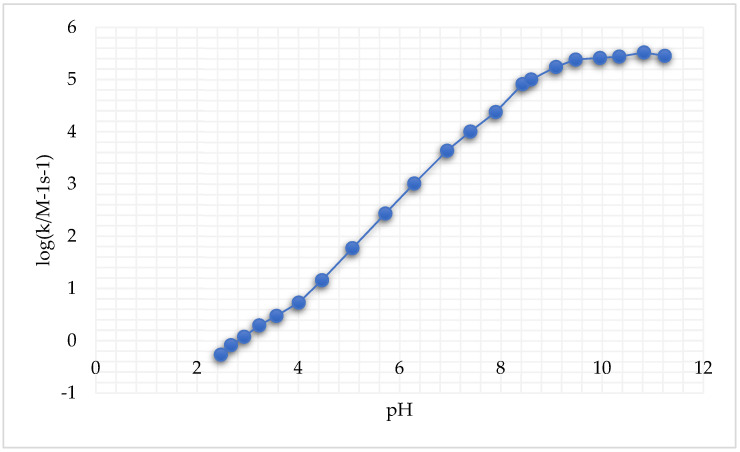
Second-order rate constants *k*′ as a function of pH at 25.0 °C and *μ* = 1.0 M for the reduction of cis,trans-[Pt(cbdca)(NH_3_)_2_Cl_2_] by GSH (adapted from [246]).

A large pH interval for both cysteine species and GSH reduction reactions was considered. It was reported that these reactions, following second-order kinetics, exhibited an important increase in k′ (i.e., the rate constant of the reaction, measured in M-1s-1 for a second-order reaction) with the increase in pH (Figure 4) [246].

**Figure 5:**
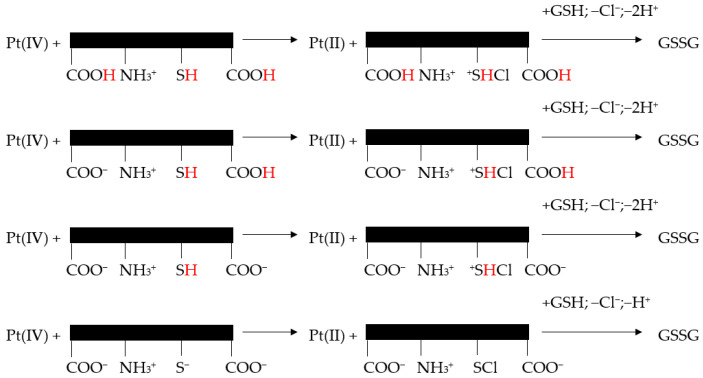
Reaction mechanism proposed for the reduction of cis, trans-[Pt(cbdca)(NH3)2Cl2] by GSH (adapted from [246]).

It appears that the reaction mechanism of Pt(IV) reduction to Pt(II) depended on the protonation level of GSH (Figure 5).

Nevertheless, all proteolytic species of cysteine/GSH simultaneously reacted with one of the trans-dichloride ligands of the Pt(IV) complex. Pt(IV) center lost Cl^+^ to the sulfur atoms and chlorothiol and sulfenylchloride were formed. Subsequently, these transient species induced the oxidation of cysteine to cystine and GSH to GSSG [246].

### 8.2. Reaction of Pt(IV) with AA

A 2012 study analyzed the reduction of Pt(IV) to Pt(II) by AA [85]. The reaction was observed at the UV-VIS spectral scan after 24 h, when two new peaks at 341 nm and 389 nm appeared corresponding to a square-planar Pt(II) complex instead of the initial 457 nm one. L-AA was used at a concentration of L-AA in the range of 0.05 to 0.3 mol dm^−3^ at a H^+^ of 0.14 mol dm^−3^ and I = 0.5 mol dm^−3^. As a result of these observations, the authors classified the reaction as a reductive elimination reaction. Another spectral assay that proved a rapid reductive elimination reaction is the formation of dehydroascorbate along with Pt(II) from the Pt(IV) halide complex reduced by AA [85].

## 9. Conclusions and Prospective

This manuscript reviewed the results of an array of studies on the most important properties of Pt(IV)-based complexes in cancer management. Given the close link between OS and cancer initiation and progression and the fact that Pt(IV) complexes are intracellularly reduced to Pt(II) complexes by GSH and AA, besides their role as prodrugs in cancer treatment, Pt(IV)-based complexes could be used in evaluating the antioxidant capacity of serum, and by that, increase the range of usage of Pt(IV) complexes in medicine. Integrating these data into a larger panel of cancer biomarkers could add more information regarding Pt(IV) mechanisms of action, and by that, improve the cancer approach in terms of screening and treatment. However, future studies are needed to experimentally prove these suppositions.

## Data Availability

No new data were created in this study.

## Abbreviations

The following abbreviations are used in this manuscript:

Pt	platinum
OS	oxidative stress
DNA	Deoxyribonucleic acid
ncRNAs	non-coding Ribonucleic acid
ROS	reactive oxygen species
miRNA	micro-Ribonucleic acid
FR	free radicals
GSH	reduced glutathione
AA	ascorbic acid
UA	uric acid
L-AA	L-ascorbic acid
NfkB	Nuclear factor-kappa B
MAPK	mitogen-activated protein kinase
ERCC	Excision Repair Cross-Complementation group
XP	xeroderma pigmentosum complementation group
UV-Vis	Ultraviolet-visible spectroscopy
LMCT	ligand-to-metal charge transfer
195Pt NMR	platinum-195 nuclear magnetic resonance
CAT	catalase
SOD	superoxide dismutase
GSSG	oxidized form of glutathione
PtNPs	platinum nanoparticles
NADPH	Nicotinamide adenine dinucleotide phosphate
Bcl-2	B-cell leukemia/lymphoma 2 protein
PARP	Poly-ADP-ribose-polymerase
